# Development of a TaqMan polymerase chain reaction detection method for the precise identification and quantification of an attenuated *Eimeria maxima* vaccine strain in poultry

**DOI:** 10.3389/fvets.2024.1397166

**Published:** 2024-05-22

**Authors:** Haozhan Jin, Haiming Cai, Shenquan Liao, Nanshan Qi, Juan Li, Minna Lv, Xuhui Lin, Junjing Hu, Yongle Song, Yibin Zhu, Xiangjie Chen, Lijun Yin, Xiaohui Zhang, Jianfei Zhang, Xu Zhang, Mingfei Sun

**Affiliations:** ^1^Key Laboratory of Livestock Disease Prevention of Guangdong Province, Key Laboratory of Avian Influenza and Other Major Poultry Diseases Prevention and Control, Ministry of Agriculture and Rural Affairs, Institute of Animal Health, Guangdong Academy of Agricultural Sciences, Guangzhou, China; ^2^School of Life Science and Engineering, Foshan University, Foshan, China

**Keywords:** avian coccidiosis, *Eimeria maxima*, attenuated live oocyst vaccines, molecular marker, TaqMan PCR

## Abstract

Avian coccidiosis, a parasitic disease prevalent in poultry, is caused by *Eimeria* species and leads to significant economic losses. The use of attenuated live oocyst vaccines has been adopted as an alternative to the use of anticoccidial drugs. However, the accurate detection and differentiation of vaccine strains from virulent ones remain challenging. Therefore, this study presents a novel TaqMan polymerase chain reaction (PCR) detection method that offers enhanced sensitivity, specificity, and reproducibility compared with traditional PCR techniques. Through whole-genome resequencing and bioinformatics analysis, we identified a molecular marker gene, Em_marker6, with a unique 21-base pair deletion specific to the *Eimeria maxima* attenuated vaccine strain. Optimized primers and probes targeting this marker enabled rapid quantification cycle value achievement and high fluorescence intensity. The standard curve’s slope of −3.540 and correlation coefficient of 0.9971 confirmed precise quantification capabilities. The TaqMan PCR method detected as few as 30 plasmid DNA copies and 50 oocysts per reaction, outperforming traditional PCR techniques by an order of magnitude. No cross-reactivity was observed with other *E. maxima* wide-type strains or common intestinal pathogens, ensuring the exclusive detection of the *E. maxima* EMPY vaccine strain. Weekly testing over 3 weeks demonstrated minimal variability, indicating robust consistency in the method’s application. Testing on 61 clinical samples revealed a 57.38% positivity rate for *E. maxima* species and 13.11% for the vaccine strain. The Em_marker6 gene exhibited genetic stability across multiple generations, confirming the detection method’s robust stability for the attenuated *E. maxima* vaccine strain. This study significantly advances the field of avian coccidiosis research and control by providing a valuable tool for monitoring vaccine purity and preventing inadvertent infections in vaccinated flocks, aligning with global efforts to curb antibiotic use in animal feed.

## 1 Introduction

Avian coccidiosis, a parasitic disease endemic to poultry, is caused by apicomplexan parasites of the genus *Eimeria* (1). Pathogenic species, such as *Eimeria acervulina*, *Eimeria maxima*, and *Eimeria tenella*, contribute to significant economic losses in the poultry industry (2). *E. maxima*, in particular, is known for its predilection for the jejunum section of the small intestine (3), although it can spread to other intestinal regions during severe infections. The effects of *E. maxima* are multifaceted, including diarrhea, intestinal wall thickening, and impaired nutrient absorption (3, 4), which can significantly impact the health and productivity of the infected poultry. Moreover, the subclinical effects of *E. maxima*, such as reduced weight gain and decreased feed conversion efficiency, can be just as damaging to the poultry industry (5). Globally, avian coccidiosis is estimated to cause annual losses exceeding £10.4 billion (6), underscoring its impact on food security and animal welfare. Therefore, the prevention and control of avian coccidiosis, especially *E. maxima*, should not be overlooked.

In response to growing concerns regarding antibiotic resistance and policies advocating for antibiotic-free animal husbandry, attenuated live oocyst vaccines have increasingly been adopted in poultry farming practices, especially in China (7). However, advancements in diagnostic technologies for these vaccines have been limited to viral and bacterial diseases. Real-time polymerase chain reaction (RT-PCR) methods have been developed to detect viral pathogens, including rotaviruses and classical swine fever virus (8–12), while the PCR detection techniques for the *Mycobacterium bovis* Bacillus Calmette–Guérin and *Brucella abortus* vaccine strains have undergone innovations (13–15). Conversely, a conspicuous gap remains in the diagnostic technologies for attenuated live antiparasitic vaccines.

Current diagnostic modalities for avian coccidiosis include conventional PCR, restriction fragment length polymorphism, recombinase polymerase amplification, and quantitative PCR (qPCR) (16–19). Despite these efforts, a critical void exists regarding effective attenuated live oocyst vaccine typing, which is requisite to preventing contamination and ensuring the efficacy of vaccination programs. Frequent avian coccidiosis outbreaks, despite extensive vaccination, highlight the imperativeness of the precise detection and quality control of vaccine strains.

This study addresses the aforementioned challenges by leveraging next-generation sequencing technology to sequence the entire genome of *E. maxima*. Through comprehensive bioinformatics analysis, we identified specific target genes that could be utilized in the development of a novel TaqMan PCR detection method. Ultimately, this approach aimed to provide a robust and sensitive means of detecting and quantifying the attenuated vaccine strain content, thereby significantly contributing to quality control measures in vaccine production and the surveillance of vaccinated chicken flocks. The development of this novel TaqMan PCR assay not only fills a critical diagnostic niche but also holds promise for expediting disease diagnosis and aiding epidemiological investigations.

## 2 Materials and methods

### 2.1 Parasitic and bacterial strains

The *E. tenella* ETGZ strain, *E. necatrix* ENHZ strain, *E. acervulina* EAGZ strain, and *E. maxima* EMPY strain, as well as *E. maxima* EMGD strain, *E. tenella* ETGD strain, *E. necatrix* ENGD strain, *E. acervulina* EAGD strain, and wild-type and precocious strains of *E. brunetti*, were isolated, identified, and preserved by the parasitology laboratory of the Institute of Animal Health at the Guangdong Academy of Agricultural Sciences. Among these, the *E. tenella* ETGZ strain, *E. necatrix* ENHZ strain, *E. acervulina* EAGZ strain, and *E. maxima* EMPY strain are components of the commercially available live attenuated coccidiosis quadrivalent vaccine (20). The sporulation and purification of chicken coccidia oocysts were performed as previously described in our published article (21). All parasitic strains were maintained in specific pathogen-free chicks aged approximately 12 days, ensuring no prior coccidial infection. Fecal samples collected within 5–7 days post-infection underwent processing using the salt flotation technique to harvest oocysts, which were subsequently stored in a 2.5% potassium dichromate solution. The entire procedure was conducted under stringent aseptic conditions to guarantee the purity and viability of the strains. Microorganisms, including *Escherichia coli* CVCC1527 strain, *Salmonella typhimurium* CVCC527 strain, *Clostridium perfringens* D25 strain, and *Candida albicans*, were maintained in our laboratory. Prior to each experiment, bacterial strains undergo revitalization and identification procedures. The strains were streaked onto agar plates to isolate individual colonies, which were then selected for identification and further propagation using conventional microbiological methods.

### 2.2 Whole-genome resequencing and selection of specific genetic markers

Genomic DNA was extracted from pure suspensions of the *E. maxima* EMPY and EMGD strains using the QIAamp DNA Mini Kit (Qiagen, Germany). Sequencing libraries were prepared using the NEBNext^®^ Ultra^™^ DNA Library Prep Kit for Illumina^®^ (New England Biolabs, United States), according to the manufacturer’s protocol. Whole-genome resequencing was performed on an Illumina NovaSeq PE150 platform at Beijing Novogene Bioinformatics Technology Co., Ltd., aiming for approximately 200× coverage. Sequencing data were aligned with the *E. maxima* Houghton strain reference genome using bioinformatics tools. Integrative Genomics Viewer was used to select specific marker genes for the *E. maxima* EMPY strain. Six candidate markers were predicted, and PCR primers were designed for their amplification (Table 1). The specificity of these markers was confirmed via PCR amplification followed by gene sequence analysis.

**Table 1:** Primers and probe used in this study.

Primer IDs	Primer sequences (5′ → 3′)	Annealing temperature (°C)	Product size (bp)
Em-marker1_CF	CGGTAGAGACTTTCCCGGTG	57	210
Em-marker1_CR	GCTGCTGACCGTAACACAAC		
Em-marker2_CF	TGCTCAACCGTTGGCTACAA	58	209
Em-marker2_CR	GTCGTAGGCTTCCCTTGCTG		
Em-marker3_CF	ATTCAGCGTGCACAACCAAC	56	216
Em-marker3_CR	CGACAGGACTGCATTGGACT		
Em-marker4_CF	TCAGCCCTGGCAATACATGG	54	223
Em-marker4_CR	CAAGCGAAGGCGGTAAGTTG		
Em-marker5_CF	ATAATCTTCGGCGGTGCTGT	57	241
Em-marker5_CR	TGACGGTGGGTGTCACGATA		
Em-marker6_CF	GACCCAGCCAGCTACTGCTA	57	201
Em-marker6_CR	GGAACTGGAACTCTCCTCTCCTA		
Em-marker6_QF	AGCCACTTCTTGTAGGACC	56	183
Em-marker6_QR	CTCCTCTCCTATAAGAATGGTTGT		
Probe1	FAM-CTGCTGCGGCCTCCTAGCTA-TAMRA		
MAX-F	TCGTTGCATTCGACAGATTC		138
MAX-R	TAGCGACTGCTCAAGGGTTT	
MAX-P	FAM-ATTGTCCAGCCAAGGTTCCCTTCG-BHQ1	

### 2.3 Design and selection of primers and probes

**Figure 1:**
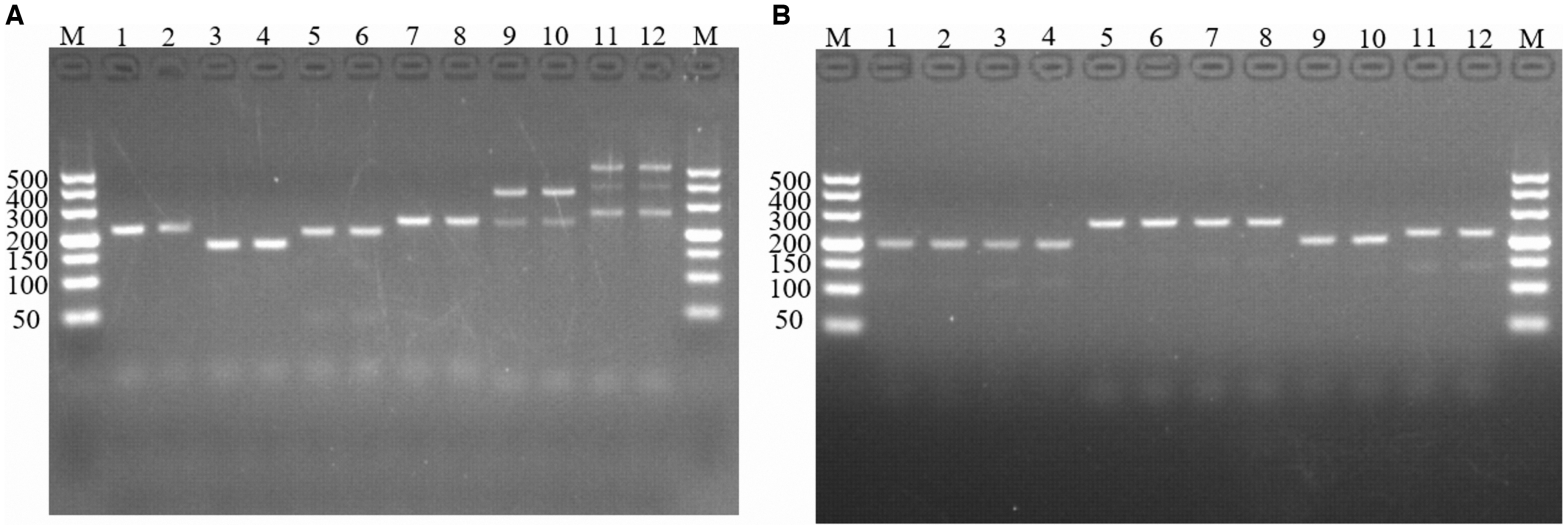
PCR amplification of molecular markers Em-marker1–6. Analysis of reaction products via agarose gel electrophoresis. **(A)** PCR amplification of molecular markers Em-marker1–3. Lanes M, 500 bp DNA ladder marker; Lanes 1–2, amplification products of the Em-marker1 primer from the EMGD strain; Lanes 3–4, amplification products of the Em-marker1 primer from the EMPY strain; Lanes 5–6, amplification products of the Em-marker2 primer from the EMGD strain; Lanes 7–8, amplification products of the Em-marker2 primer from the EMPY strain; Lanes 9–10, amplification products of the Em-marker3 primer from the EMGD strain; Lanes 11–12, amplification products of the Em-marker3 primer from the EMPY strain. **(B)** PCR amplification of molecular markers Em-marker4–6. Lanes 1–2, amplification products of the Em-marker4 primer from the EMGD strain; Lanes 3–4, amplification products of the Em-marker4 primer from the EMPY strain; Lanes 5–6, amplification products of the Em-marker5 primer from the EMGD strain; Lanes 7–8, amplification products of the Em-marker5 primer from the EMPY strain; Lanes 9–10, amplification products of the Em-marker6 primer from the EMGD strain; Lanes 11–12, amplification products of the Em-marker6 primer from the EMPY strain.

Sequence alignment of the specific marker genes from the *E. maxima* EMPY and EMGD strains was performed using DNAMAN software (Lynnon Biosoft, United States) (Figure 1). Primers targeting the entire length of the six specific marker genes were designed based on conserved regions (Table 1). After PCR identification and sequencing analysis, the Em_marker6 gene was selected for further analysis. TaqMan PCR primers and probes were designed to differentiate the EMPY strain from the EMGD strain based on the Em_marker6 gene’s insertion region (Table 1). The probe was labeled FAM at the 5′ end and TAMRA at the 3′ end (Table 1). Moreover, species-specific identification primers, MAXF/MAXR/MAX-P (19), were employed as an internal amplification control. All primers and probes were synthesized by Guangzhou Aijibio Technology Co., Ltd.

### 2.4 Recombinant plasmid construction

Genomic DNA from the *E. maxima* EMPY and EMGD strains was extracted using the E.Z.N.A.^®^ Stool DNA Kit (Omega, GA). The target gene was amplified via PCR using the following reaction mix: 25 μL of TaKaRa Taq^™^ Version 2.0 plus dye, 1 μL each of the upstream and downstream primers (Em-marker6_CF and Em-marker6_CR, 10 μmol/L), 21 μL of double-distilled water (ddH_2_O), and 2 μL of DNA template. The thermal cycling conditions were as follows: initial denaturation at 94°C for 5 min; denaturation at 94°C for 30 s, annealing at 55°C for 30 s, and extension at 72°C for 30 s over 35 cycles; and final extension at 72°C for 10 min. PCR products were analyzed using 3% agarose gel electrophoresis and purified using a gel extraction kit. Purified fragments were cloned into the pMD18-T vector and sequenced by Guangzhou Aijibio Technology Co., Ltd. Recombinant plasmids containing the target gene from both the *E. maxima* EMPY and EMGD strains were used to construct the TaqMan PCR standard curve and evaluate assay reproducibility.

### 2.5 Establishment and optimization of the TaqMan PCR method

The initial TaqMan PCR reaction system and program were established according to the instructions of the Premix Ex Taq^™^ (Probe qPCR) (TaKaRa, Japan). The final optimized reaction conditions, including additives such as dimethyl sulfoxide (DMSO), were determined empirically.

### 2.6 Establishment of the TaqMan PCR standard curve

The DNA concentration of the recombinant plasmid (pMD18-Em_marker6) was measured using a Micro Drop ultramicro spectrophotometer (BIO-DL, China), and the copy number was calculated according to previous study (22). A serial dilution series ranging from 10^1^ to 10^8^ copies/μL was prepared and stored at −40°C to serve as a standard for the TaqMan PCR assay. The reaction mix (10 μL) contained 5 μL of 2× Premix Ex Taq^™^ (Takara, Japan), 0.5 μL each of the upstream and downstream primers (Em-marker6_QF1 and Em-marker6_QR1, 10 μmol/L), 0.8 μL of the probe (2.5 μmol/L), 1 μL of 40% DMSO, 0.2 μL of ddH_2_O, and 2 μL of DNA template. Real-time PCR was performed using a CFX96^™^ Real-Time System (Bio-Rad, United States) under the following conditions: initial denaturation at 95°C for 3 min, denaturation at 95°C for 10 s and annealing at 60°C for 30 s over 40 cycles, and fluorescence detection at 60°C.

### 2.7 Validation of the TaqMan PCR method

#### 2.7.1 Sensitivity assessment

To evaluate the sensitivity of the TaqMan PCR method, serial dilution was performed using the recombinant plasmid pMD18-Em_marker6 and genomic DNA from the *E. maxima* EMPY strain. The plasmid DNA was diluted from 1:10^2^ to 1:10^9^, corresponding to a range of 3.0 × 10^8^–3.0 × 10^1^ copies per reaction. Similarly, genomic DNA was diluted from 1:10^0^ to 1:10^5^, equivalent to 5.0 × 10^5^–5 oocysts per reaction. Each dilution was subjected to triplicate TaqMan PCR analyses to ensure consistency and accuracy in quantification. The limit of detection was determined as the lowest concentration at which all three replicates were positive.

#### 2.7.2 Specificity evaluation

The specificity of the TaqMan PCR method was assessed by testing against various *Eimeria* strains, including the *E. maxima* EMPY, *E. maxima* EMGD, *E. tenella* ETGZ, *E. tenella* ETGD, *E. necatrix* ENHZ, *E. necatrix* ENGD, *E. acervulina* EAGZ, *E. acervulina* EAGD, and *E. brunetti* wild-type strains. Additionally, common enteric bacterial pathogens, such as *E. coli*, *S. typhimurium*, *C. perfringens*, and *C. albicans*, were included to identify potential cross-reactivity. Bacterial DNA samples were extracted using the Bacterial Genome DNA Extraction Kit (Tiangen, China). The concentration and purity of DNA were determined using the Micro Drop Ultra Micro Spectrophotometer (Bio-DL, China). All samples were tested in duplicate, and the results were confirmed using gel electrophoresis and sequencing analysis where necessary.

#### 2.7.3 Reproducibility analysis

To determine the reproducibility of the TaqMan PCR method, three independent experiments were conducted over a 1-week period. Each experiment entailed the use of recombinant plasmid DNA, genomic DNA from the *E. maxima* EMPY strain, and genomic DNA from the *E. maxima* EMGD strain. The cycle threshold (Ct) values obtained from each experiment were recorded and data analyzed using Microsoft Excel 2022. The mean Ct value and standard deviation (SD) were calculated for each set of replicates. The coefficient of variation (CV) was subsequently computed to assess the level of consistency across the different experimental batches. A CV value below 5% was considered acceptable for high reproducibility.

### 2.8 Clinical samples test verification

A total of 61 fecal samples suspected of *E. maxima* infection were collected from chicken farms across 11 provinces in China, including Anhui, Fujian, Guangdong, Guangxi, Hebei, Hunan, Jiangsu, Jiangxi, Sichuan, Yunnan, and Zhejiang. And eight samples originated from a chicken farm with a history of inoculating a commercial live coccidiosis quadrivalent vaccine, which showed no clinical symptoms. The oocyst samples from eight successive generations of the *E. maxima* EMPY strain were gathered during animal experiments to assess the genetic stability of Em_marker6 and the reliability of TaqMan PCR. Genomic DNA was extracted from all fecal samples using the E.Z.N.A.^®^ Stool DNA Kit. The samples were then subjected to *E. maxima* species-specific TaqMan PCR and attenuated *E. maxima* vaccine strain TaqMan PCR. Ultimately, all products testing positive for the *E. maxima* vaccine were sequenced by Guangzhou Aijibio Technology Co., Ltd.

## 3 Results

### 3.1 Screening and identification of molecular marker in attenuated *Eimeria maxima* vaccine strain

**Figure 2:**
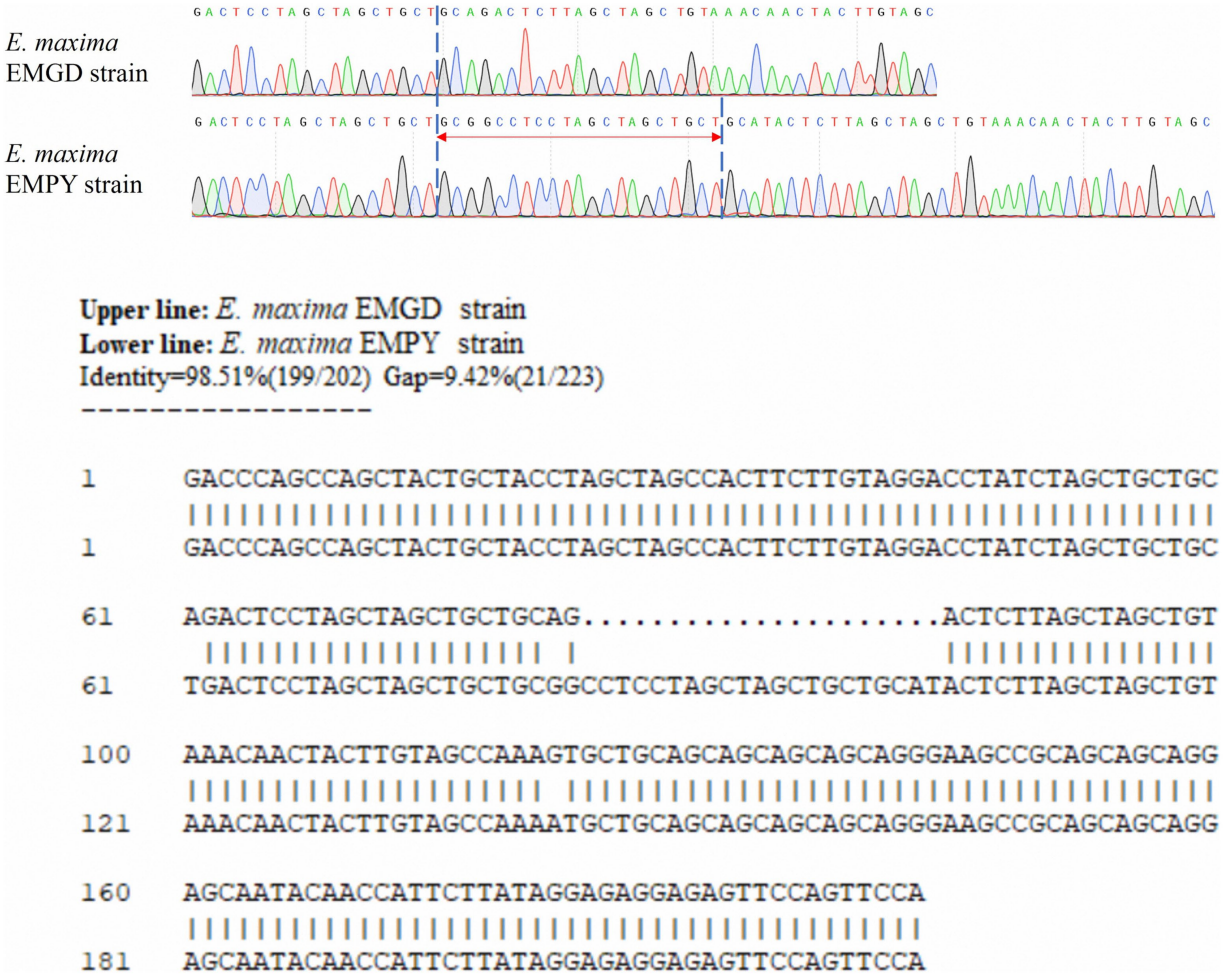
Alignment of partial sequences of the Em_marker6 gene from the *E. maxima* EMGD and EMPY strains.

Six molecular marker genes were successfully predicted using bioinformatics analysis (Supplementary Figure S1). They were subjected to PCR amplification and electrophoresis (Figure 1), followed by the sequencing of single-banded PCR products. The sequencing results revealed that the Em_marker6 in vaccine strain possessed an additional 21 base pairs compared with the precocious strain (Figure 2).

### 3.2 Amplification and standard curves of attenuated *Eimeria maxima* vaccine strain TaqMan PCR method

After optimizing the TaqMan PCR reaction system and program, as detailed in section 2.5, we achieved rapid and stable Ct values with optimal fluorescence intensity. The optimized conditions included a primer concentration of 500 nM, probe concentration of 200 nM, and the inclusion of 4% DMSO. Deviations from these concentrations resulted in delayed Ct values and reduced detection sensitivity, as illustrated in Supplementary Figure S2.

**Figure 3:**
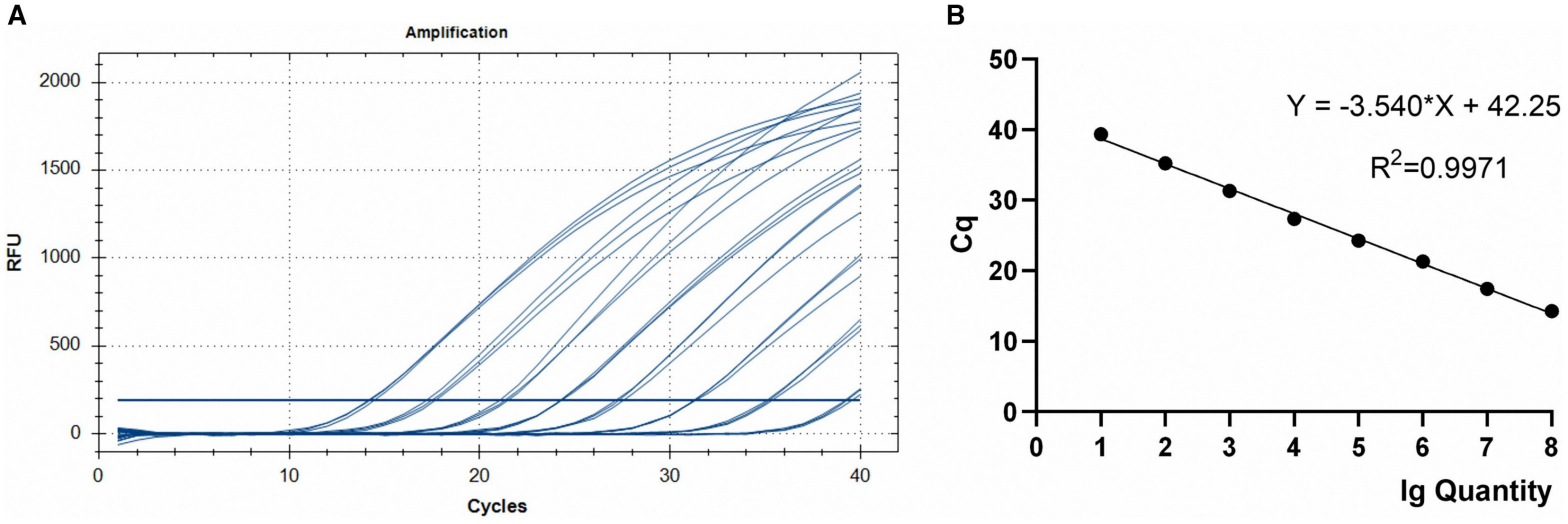
TaqMan PCR amplification **(A)** and standard **(B)** curves for the 10^8^–10^1^ copies of the Em_marker6 gene in plasmid pMD18-Em_marker6.

Using serial dilutions of recombinant plasmid DNA from the *E. maxima* EMPY strain, a standard curve was constructed, yielding a slope of −3.540 and correlation coefficient of 0.9971 (Figure 3). This strong correlation between Ct values and template concentrations enabled precise quantification of each sample’s oocyst content.

### 3.3 Sensitivity and specificity of attenuated *Eimeria maxima* vaccine strain TaqMan PCR method

**Figure 4:**
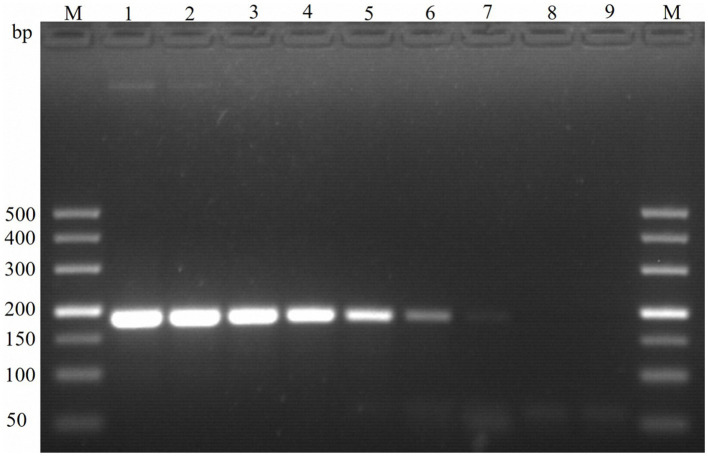
Sensitivity of PCR detection of the molecular marker Em-marker6. Lanes M, 500 bp DNA ladder marker; Lane 1, amplification product of 3.0 × 10^8^ plasmid copies; Lane 2, amplification product of 3.0 × 10^7^ plasmid copies; Lane 3, amplification product of 3.0 × 10^6^ plasmid copies; Lane 4, amplification product of 3.0 × 10^5^ plasmid copies; Lane 5, amplification product of 3.0 × 10^4^ plasmid copies; Lane 6, amplification product of 3.0 × 10^3^ plasmid copies; Lane 7, amplification product of 3.0 × 10^2^ plasmid copies; Lane 8, amplification product of 3.0 × 10^1^ plasmid copies; Lane 9, negative control.

The sensitivity of the TaqMan PCR technique was determined using serial dilutions of both recombinant plasmid DNA and genomic DNA from the *E. maxima* EMPY strain. The detection limit was established at a plasmid dilution of 1:10^9^, corresponding to 30 copies per reaction, while traditional PCR techniques could only detect 300 copies per reaction (Table 2). This indicates that the novel TaqMan PCR method is approximately 10 times more sensitive than traditional PCR techniques, as shown in Figure 4.

**Table 2:** Sensitivity of the TaqMan PCR method.

Plasmid	Copy number	Ct	Genomic DNA	Oocyst number	Ct
pMD18-Em_marker6	3.0 × 10^8^	14.28	EMPY strain	5.0 × 10^5^	24.31
	3.0 × 10^7^	17.46		5.0 × 10^4^	27.80
	3.0 × 10^6^	21.29		5.0 × 10^3^	31.01
	3.0 × 10^5^	24.27		5.0 × 10^2^	34.38
	3.0 × 10^4^	27.34		5.0 × 10^1^	38.07
	3.0 × 10^3^	31.30		5.0 × 10^0^	None
	3.0 × 10^2^	35.22			
	3.0 × 10^1^	39.35			
	3.0 × 10^0^	None			
NTC^a^		None	NTC^a^		None

a Negative control.

**Figure 5:**
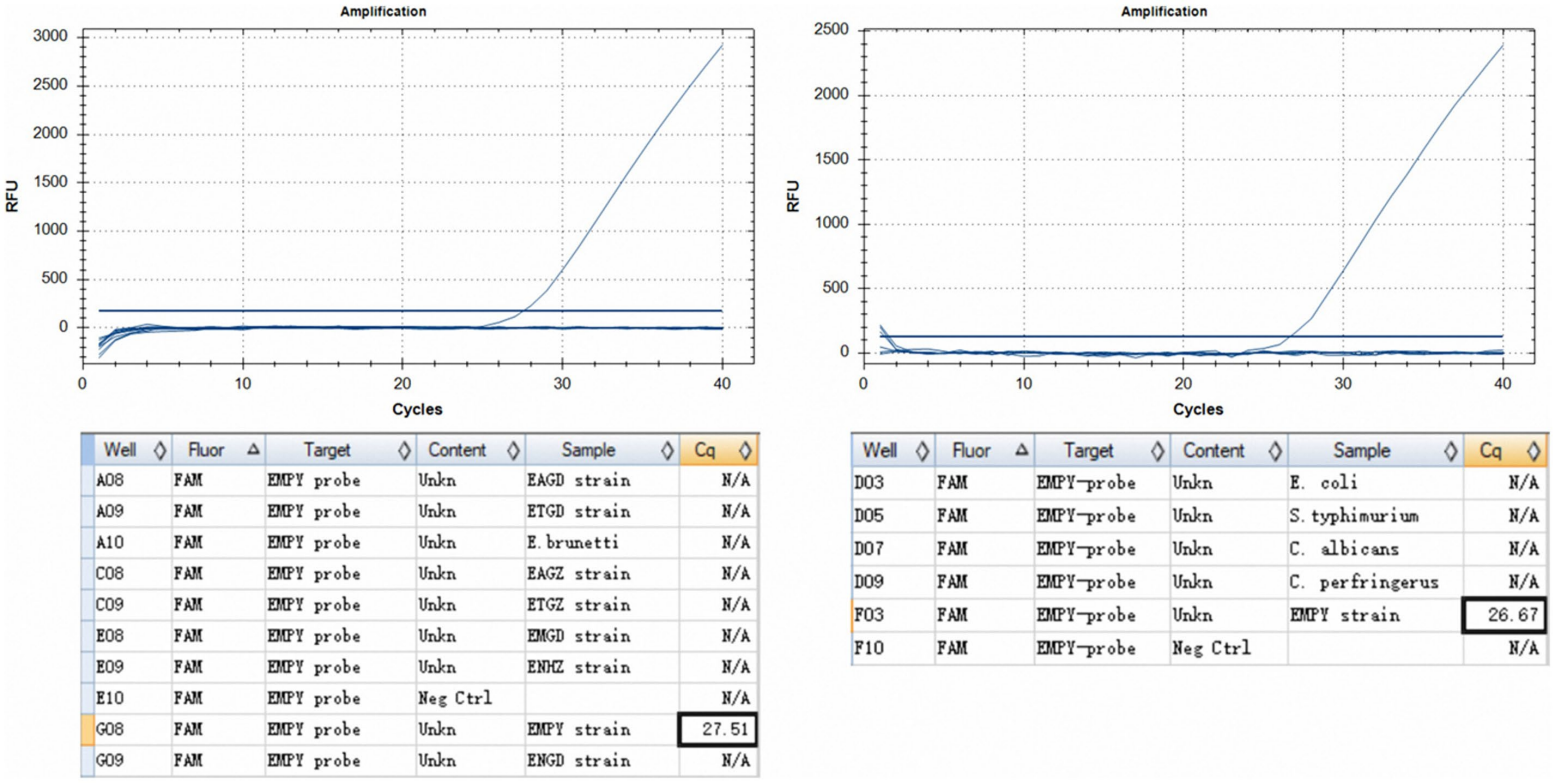
Specificity assessment of the TaqMan PCR method. **(A)** Inter-specific specificity evaluation of the TaqMan PCR method across various *Eimeria* spp. strains, including the *E. maxima* EMPY, *E. maxima* EMGD, *E. tenella* ETGZ, *E. tenella* ETGD, *E. necatrix* ENHZ, *E. necatrix* ENGD, *E. acervulina* EAGZ, *E. acervulina* EAGD, and *E. brunetti* wild-type strains. **(B)** Specificity evaluation of TaqMan PCR for the *E. maxima* EMPY strain relative to *S. typhimurium*, *E. coli*, *C. perfringens*, and *C. albicans*.

The TaqMan PCR technique consistently detected a minimum of 50 oocysts per reaction upon analyzing genomic DNA from the *E. maxima* EMPY strain. In multiplex detection scenarios involving various *Eimeria* strains and common enteric pathogens, the Ct value for the *E. maxima* EMPY strain was 23.55 cycles. When the same strains were tested alongside *E. coli*, *S. typhimurium*, *C. perfringens*, and *C. albicans*, the Ct value for the *E. maxima* EMPY strain was 25.36 cycles. Notably, owing to the 21-base pair deletion in the Em_marker6 gene of the *E. maxima* EMGD strain, no amplification curve was generated for this strain, indicating that the TaqMan PCR method did not detect DNA from the *E. maxima* EMGD strain or any other tested strains, except for the *E. maxima* EMPY strain (Figure 5).

### 3.4 Reproducibility of attenuated *Eimeria maxima* vaccine strain TaqMan PCR method

The TaqMan PCR technique’s reproducibility was evaluated via weekly testing of the recombinant plasmid, *E. maxima* EMPY strain, and *E. maxima* EMGD strain nucleic acids over a 3-week period. The results revealed an SD range of 0.03–0.20, with a CV of 0.10–0.95%, confirming the TaqMan PCR method’s high reproducibility (Table 3).

**Table 3:** Inter-assay CV of the TaqMan PCR method.

Plasmid (dilution ratio) and strain information	Ct			Ct (mean ± SD)	Coefficient of variation (CV), %
pMD18-Em-marker6 (10^−4^)	21.02	21.27	21.42	21.24 ± 0.20	0.95
pMD18-Em-marker6 (10^−5^)	24.16	24.11	24.14	24.14 ± 0.03	0.10
pMD18-Em-marker6 (10^−6^)	27.19	27.42	27.02	27.21 ± 0.20	0.74
*E. maxima* EMPY strain	34.03	34.09	34.12	34.08 ± 0.05	0.13
*E. maxima* EMGD strain	None	None	None	None	None
NTC^a^	None	None	None	None	None

a Negative control.

### 3.5 Evaluation of attenuated *Eimeria maxima* vaccine strain TaqMan PCR with clinical samples

To validate the reliability of the established TaqMan PCR method, a total of 61 clinical samples were subjected to this assay. The outcomes revealed a 57.38% positivity rate (35/61) for *E. maxima* species-specific TaqMan PCR and a 13.11% positivity rate (8/61) for attenuated *E. maxima* vaccine strains, as detailed in Supplementary Table S1. Among the 27 samples that were negative in the *E. maxima* vaccine strain TaqMan PCR assay, this suggests the likelihood of infection with wild-type *E. maxima* strains, which is consistent with the clinical pathologies observed. Additionally, eight successive generations of the *E. maxima* EMPY strain were collected during animal experiments. These samples underwent conventional PCR testing for Em_marker6, and the results were presented in Supplementary Figure S3, showing consistency across all generational samples compared to the reference sample of *E. maxima* EMPY strain. Furthermore, when these samples were tested using *E. maxima* species-specific TaqMan PCR and attenuated *E. maxima* vaccine strain TaqMan PCR, specific amplification results were obtained. Therefore, the selected Em_marker6 exhibited good genetic stability, and the attenuated *E. maxima* vaccine strain TaqMan PCR demonstrated robust detection stability.

## 4 Discussion

Avian coccidiosis, a significant intestinal parasitic disease affecting poultry, causes substantial economic losses within the global poultry sector (7). To date, 10 *Eimeria* species pathogenic to chickens have been identified, with *E. maxima* being a key causative strain of avian coccidiosis. The primary strategies for preventing and controlling this disease include pharmacological treatment and vaccination. However, extensive anticoccidial drug use has exacerbated issues related to drug resistance and residual contamination. Consequently, several countries have advocated for the reduction and replacement of these drugs, with some even prohibiting the inclusion of antibiotics in animal feed (16). Against this backdrop, coccidiosis vaccines have garnered considerable research and development attention. In China’s intensive farming systems, attenuated live oocyst vaccines have progressively emerged as the principal means of combating avian coccidiosis. Notwithstanding, quality control and potential wild-type strain contamination during the production of these attenuated live oocysts are critical determinants of vaccine efficacy (23). On this premise, precise detection methods that quantify the attenuated vaccine strain content during the production process are urgently required. Therefore, developing a rapid and sensitive technique that differentiates the *E. maxima* early maturation strain from its parental strains is crucial for the effective prevention and control of avian coccidiosis.

Stable molecular marker genes are essential for establishing reliable detection methods. Leveraging whole-genome resequencing technology and extensive strain testing, we aimed to develop an efficient and stable TaqMan PCR detection protocol. Several diagnostic techniques, including microscopic detection, traditional PCR, multiplex PCR, and real-time PCR targeting specific marker genes on the sequence-characterized amplified region (SCAR) as well as real-time PCR targeting the internal transcribed spacer-2 (ITS-2) region (17–19, 24), have been developed to diagnose avian coccidiosis. Although mature and stable TaqMan PCR detection methods based on the SCAR and ITS-2 regions have been established, these primarily identify *Eimeria* spp. without distinguishing between vaccine and virulent strains. In this study, we developed a novel TaqMan PCR detection method based on bioinformatics analysis to differentiate the *E. maxima* EMPY vaccine strain from virulent strains.

During the development of this method, we optimized the TaqMan probes and primers designed from the Em_marker6 gene to ensure the rapid and stable attainment of the Ct value and optimal fluorescence intensity. Ultimately, one probe and one primer pair were selected based on the strong correlation between Ct values and template concentrations as well as the method’s high specificity and amplification efficiency. The standard curve generated in this study had a slope of −3.540 and correlation coefficient of 0.9971, indicating that our TaqMan PCR technique can specifically detect the *E. maxima* EMPY strain without cross-reacting with other *E. maxima* virulent strains or common pathogenic bacteria found in the chicken intestine. This correlation coefficient aligns with those reported by Vrba et al. (19), who established TaqMan PCR techniques for detecting seven *Eimeria* spp., and by Gautam et al. (8), who developed real-time RT-PCR assays for detecting Rotarix^®^ and RotaTeq^®^ vaccine and wild-type strains of rotaviruses, all exhibiting correlation coefficients above 0.99. Therefore, the standard curve established in this study is accurate and reliable for quantitative analysis. The TaqMan PCR technique developed in this study is capable of specifically detecting other *E. maxima* attenuated vaccine strain (data not show) without cross-reacting with other virulent strains of *E. maxima* or common pathogens found in the chicken intestine.

Sensitivity is a crucial metric for evaluating detection methods. In this study, we determined the sensitivity of the TaqMan PCR method using serial dilutions of recombinant plasmid DNA (pMD18-Em_marker6) and genomic DNA from the *E. maxima* EMPY strain. The detection limit of the TaqMan PCR method was 30 copies per reaction compared with 300 copies generated by traditional PCR techniques (Table 2), indicating that our TaqMan PCR method is 10 times more sensitive. For genomic DNA from the *E. maxima* EMPY strain, the detection limit was 50 oocysts per reaction, exhibiting consistency with the lower limit achieved by Vrba et al. (19) using inter-species real-time fluorescence qPCR based on the SCAR region. Comparatively, He et al. (25) established a multiplex RT-PCR assay for swine epidemic diarrhea virus vaccine and wild-type strains, with a plasmid sensitivity detection limit of 1.51 × 10^4^ copies/μL, highlighting our method’s heightened sensitivity. Therefore, our method can be integrated with existing techniques for quantitative and contamination analyses of the *E. maxima* EMPY vaccine strain. On using the TaqMan PCR method to simultaneously detect the *E. maxima* EMPY vaccine strain, other *Eimeria* species, and common avian intestinal pathogens, the *E. maxima* EMPY vaccine strain exclusively exhibited amplification curves, demonstrating the method’s specificity.

The genetic characteristics of molecular markers are crucial in determining the stability of TaqMan PCR detection methods (18). In this study, we examined samples of the *E. maxima* EMPY strain over eight consecutive generations and found that the Em_marker6 molecular marker demonstrated good genetic stability throughout the continuous passage of the vaccine strain. The molecular markers used in the TaqMan PCR technique for detecting seven species of *Eimeria* spp. SCAR gene are single-copy and have not been found to exhibit intraspecific sequence variation, which ensures the stability of the method (26). In the case of the multiplex RT-PCR detection technique for the vaccine and wild strains of PEDV, the molecular markers are the S gene and ORF3 gene. The S gene is one of the most variable genes in the PEDV genome and serves as a signature gene for viral genetic variation (27). The ORF3 gene is the only accessory gene in PEDV and is an important gene related to virus production and virulence. Stable variations in the ORF3 gene have been observed during continuous cell culture, which provides assurance for the stability of detection techniques (28, 29). Therefore, the genetic stability of molecular markers is essential for the establishment of reliable detection methods. Otherwise, to assess the reproducibility of the TaqMan PCR technique, we tested the recombinant plasmid DNA pMD18-Em_marker6, genomic DNA from the *E. maxima* EMPY strain, and genomic DNA from the *E. maxima* virulent strain three times over a week. The SD and CV ranges were 0.03–0.20 and 0.10–0.95%, respectively (Table 3). To further assess the stability of our method, clinical samples were tested. The results showed that the TaqMan PCR method for *E. maxima* EMPY strain can specifically detect the feces samples from poultry farms vaccinated with the *E. maxima* EMPY strain, while it cannot detect samples from poultry farms in different provinces that have not used this vaccine. This indicates that the method has good detection performance and is stable and reliable for its intended use.

## 5 Conclusion

In conclusion, this study is the first to establish a TaqMan PCR detection method that specifically identifies the *E. maxima* EMPY vaccine strain and distinguishes *E. maxima* based on bioinformatics analysis. This method is particularly suited for the production of the *E. maxima* EMPY vaccine strain and differentiation of this strain from other virulent strains in flocks immunized with the *E. maxima* EMPY vaccine.

## Data availability statement

The raw sequencing data for bacterial communities have been submitted to the National Center of Biotechnology Information (NCBI) Sequence Read Archive under accession number PRJNA1099220.

## Ethics statement

The animal study was approved by the Animal Care and Use Committee of the Institute of Animal Health, Guangdong Academy of Agricultural Sciences. The study was conducted in accordance with the local legislation and institutional requirements.

## Author contributions

HJ: Conceptualization, Data curation, Formal analysis, Investigation, Methodology, Writing – original draft, Writing – review & editing. HC: Conceptualization, Formal analysis, Methodology, Resources, Writing – original draft, Writing – review & editing. SL: Formal analysis, Funding acquisition, Project administration, Resources, Validation, Writing – review & editing, Writing – original draft. NQ: Software, Supervision, Validation, Visualization, Writing – original draft, Writing – review & editing. JL: Investigation, Supervision, Validation, Visualization, Writing – review & editing. ML: Investigation, Supervision, Validation, Visualization, Writing – review & editing. XL: Formal analysis, Methodology, Resources, Writing – original draft. JH: Conceptualization, Investigation, Software, Supervision, Validation, Writing – review & editing. YS: Conceptualization, Data curation, Formal analysis, Project administration, Writing – review & editing. YZ: Investigation, Software, Supervision, Writing – review & editing. XC: Investigation, Supervision, Validation, Writing – review & editing. LY: Project administration, Supervision, Validation, Writing – review & editing. XiZ: Investigation, Writing – review & editing. JZ: Investigation, Project administration, Supervision, Validation, Writing – review & editing. XuZ: Formal analysis, Funding acquisition, Investigation, Methodology, Project administration, Writing – original draft, Writing – review & editing. MS: Formal analysis, Funding acquisition, Investigation, Methodology, Project administration, Resources, Writing – original draft, Writing – review & editing.
