# Supplementary material for: Development of a TaqMan polymerase chain reaction detection method for the precise identification and quantification of an attenuated Eimeria maxima vaccine strain in poultry
Source: Front Vet Sci. 2024 May 22;11:1397166. doi: 10.3389/fvets.2024.1397166 (PMC11151167; doi:10.3389/fvets.2024.1397166)
Supplement: Supplementary file 1 [file Data_Sheet_1.docx]

**Supplement Files:**

**Development of a TaqMan PCR Detection Method for the Precise Identification and Quantification of an Attenuated *E. maxima* Vaccine Strain in Poultry**

Haozhan Ji ^1,2#^, Haiming Cai ^2#^, Shenquan Liao ^2^, Nanshan Qi ^2^, Juan Li ^2^, Minna Lv ^2^, Xuhui Lin ^2^, Junjing Hu ^2^, Yongle Song ^2^, Yibin Zhu ^2^, Xiangjie Chen ^2^, Lijun Yin ^2^, Xiaohui Zhang ^2^, Jianfei Zhang ^2^, Xu Zhang ^1*^, Mingfei Sun ^2*^.

^1^ School of Life Science and Engineering, Foshan University, Foshan 528231, China

^2^ Key Laboratory of Livestock Disease Prevention of Guangdong Province, Key Laboratory of Avian Influenza and Other Major Poultry Diseases Prevention and Control, Ministry of Agriculture and Rural Affairs, Institute of Animal Health, Guangdong Academy of Agricultural Sciences, Guangzhou, 510640, China.

# These authors contributed equally.

* Corresponding author: Prof. Mingfei Sun; Dr. Xu Zhang.

E-mail address: [smf7810@126.com](mailto:smf7810@126.com), (Prof. Mingfei Sun); 123zhxpost@163.com, (Dr. Xu Zhang).


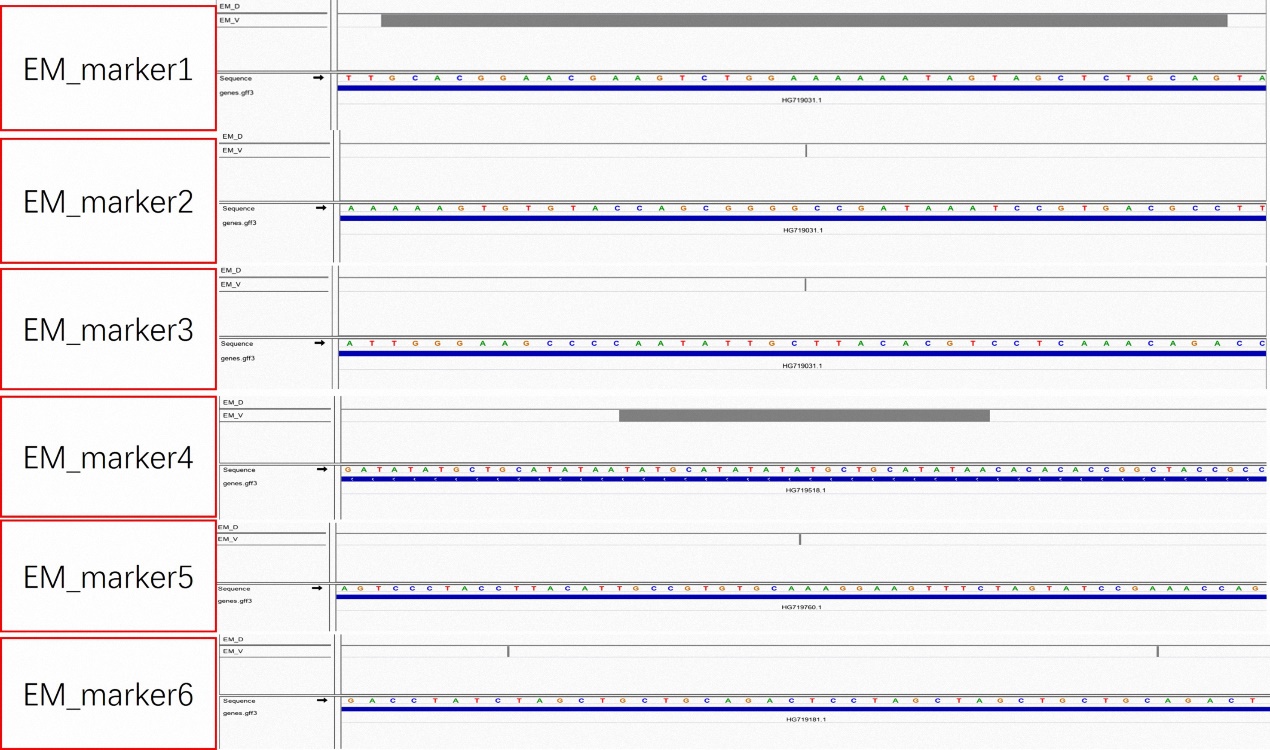


**Figure S1**. Multiple Sequence Alignment of Em_marker1 to Em_marker6. Sequence alignment results depicting the comparison of Em_marker1 through Em_marker6. The aligned sequences illustrate the homology and variations among the markers. Conserved regions and potential variations across the markers are highlighted, providing insights into the sequence similarities and differences. This alignment serves as a comprehensive overview for understanding the genetic relationships and structural features of the analyzed markers.


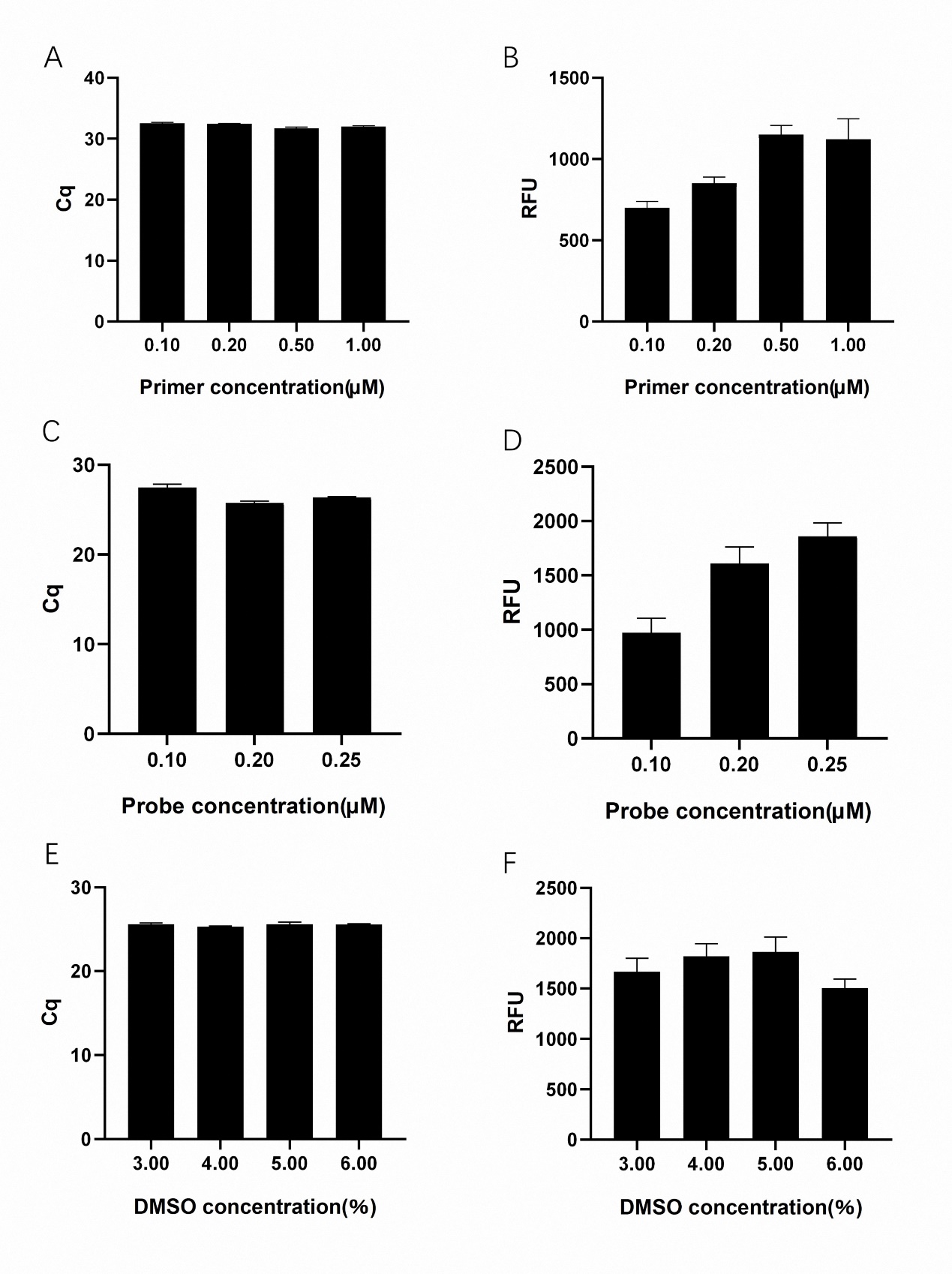


**Figure S2**. Optimization results of TaqMan PCR primers, probes, and DMSO concentration. With Cq value as the primary parameter and RFU value as the auxiliary parameter, the optimal primer concentration of 0.5 was obtained by optimizing the primer concentration, probe concentration, and DMSO concentration μ M; Optimal probe concentration 0.2 μ M; The optimal DMSO percentage is 4%.


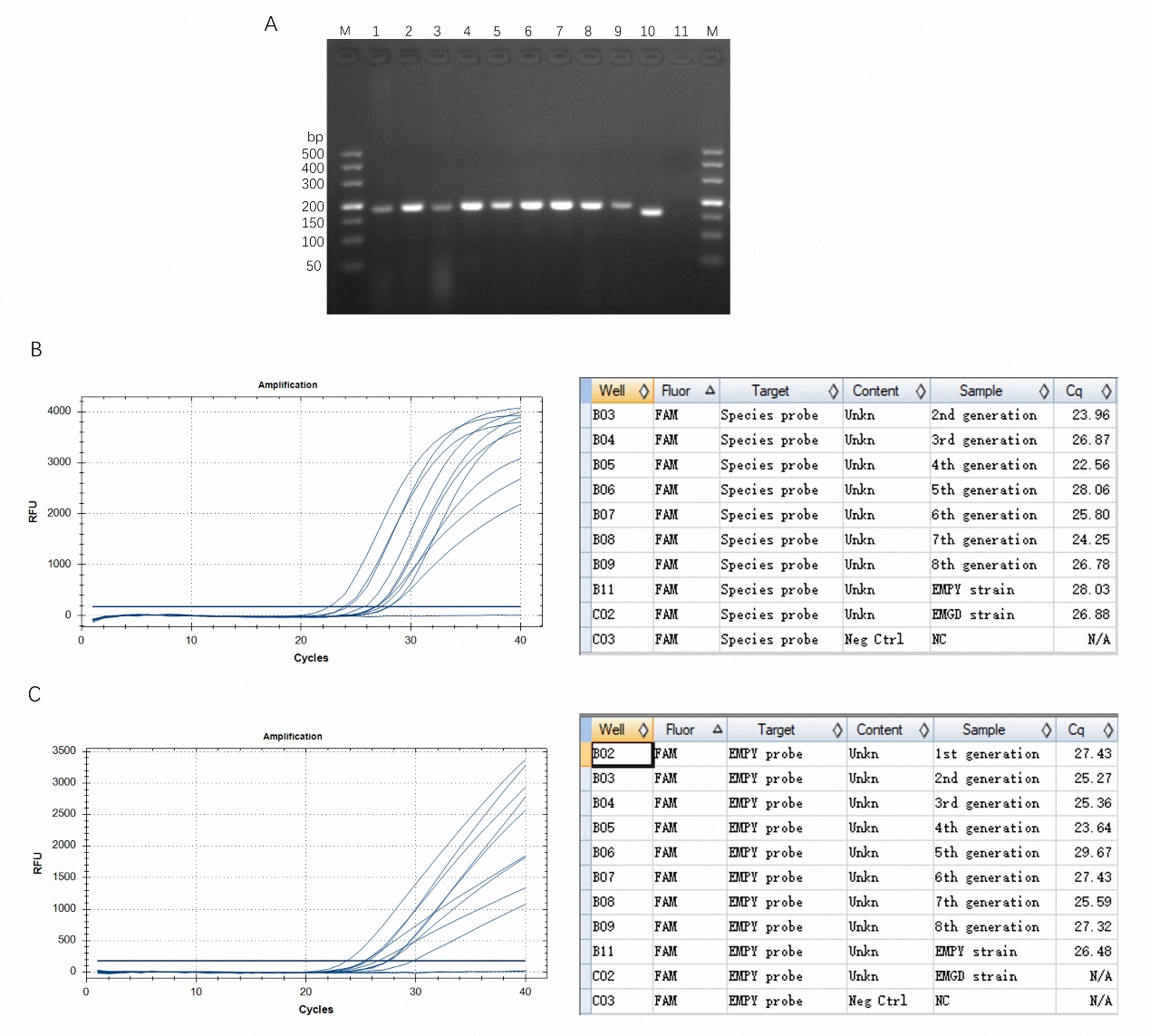


Figure S3. The stability of Em_marker6 detection and the TaqMan PCR method across eight consecutive passages of *Eimeria maxima* EMPY strain samples. (A) PCR identification results of Em_marker6 detected in the eight generation. Lane M represents the DL500 DNA Marker, lanes 1-8 display PCR products of the Em_marker6 gene using specific primers for each passage, lane 9 depicts the PCR product of the Em_marker6 gene for the *E. maxima* EMPY strain, lane 10 shows the PCR product of the Em_marker6 gene for the *E. maxima* EMGD strain, and lane 11 serves as the negative control. (C) Results of TaqMan PCR detection using *E. maxima* species-specific primer pairs (MAXF/MAXR/MAX-P). (D) Results of TaqMan PCR detection using primers and a TaqMan probe designed for detecting the attenuated *E. maxima* vaccine strain established in this study.

Table S1. Clinical sample information and detection results

| Sample ID | District | Provinces | Vaccine ^a^ | Species Ct Values ^b^ | EMPY Ct Values ^c^ |
| --- | --- | --- | --- | --- | --- |
| 1 | Bishan-1 | Chongqing | - | N.A. | N.A. |
| 2 | Bishan-2 | Chongqing | - | N.A. | N.A. |
| 3 | Cangzhou-1 | Hebei | - | N.A. | N.A. |
| 4 | Cangzhou-2 | Hebei | - | N.A. | N.A. |
| 5 | Cangzhou-3 | Hebei | - | N.A. | N.A. |
| 6 | Chuzhou-1 | Anhui | - | 30.04 | N.A. |
| 7 | Chuzhou-2 | Anhui | - | 29.72 | N.A. |
| 8 | Dali-1 | Yunnan | - | 34.88 | N.A. |
| 9 | Dali-2 | Yunnan | - | 37.27 | N.A. |
| 10 | Dazhou-1 | Sichuan | - | N.A. | N.A. |
| 11 | Dazhou-2 | Sichuan | - | N.A. | N.A. |
| 12 | Deyang-1 | Sichuan | - | 34.09 | N.A. |
| 13 | Deyang-2 | Sichuan | - | 33.22 | N.A. |
| 14 | Guangzhou-1 | Guangdong | Vaccine | 25.01 | 25.90 |
| 15 | Guangzhou-2 | Guangdong | Vaccine | 24.25 | 24.22 |
| 16 | Guangzhou-3 | Guangdong | Vaccine | 26.63 | 28.11 |
| 17 | Guangzhou-4 | Guangdong | Vaccine | 25.29 | 26.29 |
| 18 | Hengyang-1 | Hunan | - | 38.82 | N.A. |
| 19 | Hengyang-2 | Hunan | - | N.A. | N.A. |
| 20 | Hezhou-1 | Guangxi | - | 36.18 | N.A. |
| 21 | Hezhou-2 | Guangxi | - | 34.52 | N.A. |
| 22 | Huaian-1 | Jiangsu | - | 34.40 | N.A. |
| 23 | Huaian-2 | Jiangsu | - | 34.26 | N.A. |
| 24 | Huaian-3 | Jiangsu | - | N.A. | N.A. |
| 25 | Huaian-4 | Jiangsu | - | N.A. | N.A. |
| 26 | Jian-1 | Jiangxi | - | N.A. | N.A. |
| 27 | Jian-2 | Jiangxi | - | 37.58 | N.A. |
| 28 | Kunming-1 | Yunnan | - | N.A. | N.A. |
| 29 | Kunming-2 | Yunnan | - | 33.48 | N.A. |
| 30 | Kunming-3 | Yunnan | - | 35.80 | N.A. |
| 31 | Linxiang-1 | Hunan | - | 34.22 | N.A. |
| 32 | Linxiang-2 | Hunan | - | 33.32 | N.A. |
| 33 | Meizhou-1 | Guangdong | - | N.A. | N.A. |
| 34 | Meizhou-2 | Guangdong | - | N.A. | N.A. |
| 35 | Meizhou-3 | Guangdong | - | N.A. | N.A. |
| 36 | Meizhou-1 | Sichuan | - | N.A. | N.A. |
| 37 | Meizhou-2 | Sichuan | - | N.A. | N.A. |
| 38 | Meizhou-3 | Sichuan | - | N.A. | N.A. |
| 39 | Nanjing | Jiangsu | - | N.A. | N.A. |
| 40 | Nantong-1 | Jiangsu | - | N.A. | N.A. |
| 41 | Nantong-2 | Jiangsu | - | N.A. | N.A. |

Continued Table S1

| Sample ID | District | Provinces | Vaccine ^a^ | Species Ct Values ^b^ | EMPY Ct Values ^c^ |
| --- | --- | --- | --- | --- | --- |
| 42 | Putian-1 | Fujian | - | 34.19 | N.A. |
| 43 | Putian-2 | Fujian | - | 34.64 | N.A. |
| 44 | Sanmenxia-1 | Henan | - | N.A. | N.A. |
| 45 | Sanmenxia-2 | Henan | - | N.A. | N.A. |
| 46 | Sanshui-1 | Guangdong | - | 33.38 | N.A. |
| 47 | Sanshui-2 | Guangdong | - | 32.75 | N.A. |
| 48 | Shaoxing-1 | Zhejiang | - | 34.70 | N.A. |
| 49 | Shaoxing-2 | Zhejiang | - | 35.81 | N.A. |
| 50 | Wuhu-1 | Anhui | - | 38.65 | N.A. |
| 51 | Wuhu-2 | Anhui | - | N.A. | N.A. |
| 52 | Yancheng | Jiangsu | - | N.A. | N.A. |
| 53 | Yulin-1 | Guangxi | - | 33.66 | N.A. |
| 54 | Yulin-2 | Guangxi | - | 33.28 | N.A. |
| 55 | Yulin-3 | Guangxi | - | N.A. | N.A. |
| 56 | Yulin-4 | Guangxi | - | 36.68 | N.A. |
| 57 | Yunfu-1 | Guangdong | Vaccine | 26.89 | 27.91 |
| 58 | Yunfu-2 | Guangdong | Vaccine | 23.49 | 24.42 |
| 59 | Yunfu-3 | Guangdong | Vaccine | 25.36 | 25.28 |
| 60 | Yunfu-4 | Guangdong | Vaccine | 24.67 | 26.01 |
| 61 | Zhaoqing-1 | Guangdong | - | 34.24 | N.A. |

^a^ "-" indicates that the farm did not use any vaccines or relied primarily on anticoccidial drugs for treatment. "Vaccine" refers to the use of a commercially available live coccidiosis quadrivalent vaccine, containing *E. tenella* ETGZ strain, *E. necatrix* ENHZ strain, *E. acervulina* EAGZ strain, and *E. maxima* EMPY strain.

^b^ Corresponding Ct values obtained using *E. maxima* species-specific primer pairs (MAXF/MAXR/MAX-P) for TaqMan PCR detection.

^c^ Corresponding Ct values obtained using primers and TaqMan probe for the detection of the attenuated *E. maxima* vaccine strain established in this study. "N.A." stood for "No Amplification", which meant that no fluorescent signal was detected.
